# The Extirpation of the Human Milt

**Published:** 1860-02

**Authors:** O. Martini

**Affiliations:** Dresden


*The Extirpation of the Human Milt.* By O. MARTINI, M. D., of Dresden.

The lamentable inclination manifested by some to transfer upon the diseased human body any operation which proves not absolutely lethal in a healthy dog, cat, hog, or frog, cannot be considered a therapeutical aberration only, but deserves the strongest disapproval, as a murderous attempt, if gratified, where there is in reality no indication for it, and no excuse but the desire of certain men to raise themselves in that way to a somewhat elevated position in surgery. We know occurrences of that kind, which we do not hesitate to denounce as unreasonable and unnecessary injuries on the part of the physician, instead of being "a scrupulous compliance with his duty." Until it is determined whether splenotomy is entitled to any claim in surgery or not, we would remind the men referred to, of the following words, expressed by Sydenham. "Aegrorum nemo a me alias tractatus est, quam egome tractari cuperem si mihi ex iisdem morbis aegrotare contingeret."

On the other hand, our thanks are due to Gustav Simon, of Darmstadt, for the great trouble he has taken to expose, in a scientific manner, the true merits of the enthusiastically proposed new operation, extirpating the milt. In speaking of this operation, we cannot but follow the author in the arrangement presented in his work: "Die Extirpation der Miltz am Menschen nach dem jetzigen Standpuncte der Wissenschaft beurtheilt." (The extirpation of the human milt considered from the present point of view of science.) Giessen, 1857.

I. Extirpation of chronic splenetic tumors, the organ itself being in its normal place.

Ovariotomy is the only operation that may be compared with such a removal of the spleen. Kuechler (Extirpation eines' miltz tumors, mit wissenschaftlicher Beleuchtung der Frage; Darmstadt, 1858) speaks of ovariotomy as "having obtained and maintaining its place in surgery;" but it is objected to by Dieffenbach, Scanzoni, and Velpeau, on account of the danger unavoidably connected with it. Sixty-one ovariotomies performed in Germany, resulted in a mortality of more than seventy-two per cent., while the rest of those operated upon escaped without serious damages, and only about nineteen per cent. of them derived really a benefit from the operation.

Three cases where extirpation was resorted to for the removal of chronic tumors in the spleen, are on record.

Zaccarelli operated, in 1549, the wife of a Greek centurion, suffering from oppilation of the milt. That organ protruded through the wound made in the abdomen, was separated from its connection, and taken out. The patient recovered in twenty-four days. But the correctness of this case is doubted by almost all eminent surgical writers.

The second operation dates back only to 1826. The patient, a woman, at the age of twenty-two, laboring under cirrhosis of the liver and ascites, had acquired a swelling of the milt, from suppression of perspiration and menses, irrascibility, &c. Tormenting pains in the tumor and the threatening dissolution, induced Dr. Quittenbaum to undertake the removal of the spleen. Carrying the knife along the linea alba to an extent of ten inches, he severed the ligamentum gastro-lineale, ligatured the vessels without regard to the nerves, and separated the diseased organ, which weighed five pounds. Six hours afterwards the patient died from exhaustion. We cannot approve of the operation in this instance, the hopeless condition of the patient having been evident to every one.

The third case is the one related by Kuechler, in the work cited before.

The tumor was fourteen inches in length, by seven in width, weighed three pounds, and had existed for fourteen years. The patient died two hours after the operation, from effusion of blood into the abdominal cavity. The cut was made along the exterior margin of the left rectus abdominis, in a length of four inches. Seven arteries were tied, care being taken not to include the nerves. There was no urgent symptom demanding the operation, the general condition of the man appearing scarcely damaged. The patient had been under observation only five days. No explanation is given of the origin and the development of the disease; nothing stated about the treatment he had undergone through so many years, not even a microscopial analysis of the blood mentioned. The pretended cause of death may be justly doubted; the facts given rather indicate the operation itself as the cause of it.

This evidence, although quite insufficient for a final decision, allows the assumption that the extirpation of chronic splenetic tumors is always highly dangerous, if not absolutely lethal. In this regard it is at least just as dangerous to life as ovariotomy.

Kuechler denies such a degree of lethality, and ascribes to the whole operation a lesser importance, while he claims a greater facility in the performance, there being, in his judgment, scarcely any of the dtfficulties encountered in ovariotomy, the spleen offering itself as soon as the integuments are separated, and filling the wound, preventing any prolapse of the bowels, as well as the entering of air or blood. This, however, can never be prevented with certainty. A far greater wound is required; unnatural connections may interfere with the extirpation. The numerous successful operations on animals prove nothing, thousands of hogs being deprived of their ovaries without serious consequence, and still the same operation proving disastrous in by far the greatest number of instances when performed on a human being. Taking the question physiologically, Kuechler feels rather encouraged from the limited knowledge we have of the functions of the milt, and the small importance consequently attached to it. But Simon thinks the sudden abolition of that function would be more likely to increase the danger already connected with the operation, not to speak of the undecided question, if life and health can be maintained without that function. Even animals pass easier through the extirpation of the ovaries than that of the spleen, and it cannot be overlooked, that the human milt surpasses that of all the animals so far experimented on, in its proportions to the other organs of the body. Besides this, a morbidly affected milt presents other conditions by its increased size, its multiplied vessels, adhesions, &c. It can never be proved to have ceased in its function, even after protracted diseases. The facts established by the removal of the spleen in healthy persons, accidentally wounded, would perhaps justify a similar operation, when an imminent danger (fiom gangrene, effusion of blood, &c.) can be averted thereby. It is quite another case with a chronic tumor, there being no immediate danger, and the utility of the most successful operations remaining very questionable, under the present amount of our knowledge.

The only conditions that would admit the extirpation of a chronic milt tumor, are thus enumerated by Simon: No complication with disease in other organs, or in the blood; undoubted correctness of the diagnosis; immediate danger of life. But as these conditions are never existing when the spleen is morbidly increased in size, the operation is objectionable under all circumstances, except perhaps one: it might be justifiable, when a chronic affection, before it is considerably developed, and before it has undermined the strength of the patient to any degree, turns, by some accident, into an acute disease (rupture, for instance, or incarceration) so as to put life into jeopardy, and even then provided that there is no doubt in regard to the diagnosis, and the patient is able to bear the operation.

II. Extirpation of the milt, not hypertrophied but prolapsed, in consequence of penetrating wounds in its neighborhood.

Seventeen cases of this kind are known since 1581, the organ being either totally removed, or only a part of it. Not one of these cases ended fatally, rendering it highly probable that the spleen may be extirpated in a healthy person without impairing the life and health for at least some time. But notwithstanding this favorable conclusion, there is another indication to be minded. The spleen falling through an abdominal wound, ought to be replaced, if not morbidly altered or otherwise spoiled. A hemorrhage from the milt itself, which cannot be stopped, suppuration or gangrene of the same, would certainly demand the extirpation, which might even be attempted, when, under similar circumstances, the organ is retained in the abdominal cavity.

III. Extirpation of the movable spleen (or wandering milt, as the Germans call it.)

This abnormity has not been noticed until a few years ago, and the observations are consequently not very numerous, amounting in all to about fifteen. It may be congenital or acquired. Sometimes the organ is not changed, although it is generally increased in size, when dislocated.

Kuechenmeister (Letter to Prof. Dietl, "Wien Medic. Wochenschr." 27, 1856) proposes to extirpate a wandering, movable spleen, if the trouble it causes is not removed by quinine. Certainly a correct diagnosis is a *conditio sine qua non.*

According to Simon, the symptoms dependent on such a state are more distressing, and at the same time more distinct, than those connected with a tumor in the normal place. The experience of Dietl shows the inefficacy of any therapeutical remedies. Furthermore, there is no great danger in the operation, it being admissible before the tumor acquires any considerable size; hemorrhage can be easier prevented, ligatures applied more securely.

Against such an operation, however, there are many and strong objections. We cannot but consider the extirpation even of a wandering spleen, much more injurious to life than ovariotomy. Then, it is not yet proved that the removal of the spleen, under the most promising circumstances, does not result ultimately in heavy damages to health and life. Complications which would contra-indicate the operation, seldom escapes the most scrutinous examinations. All symptoms referring directly to the tumor (such as heaviness in the lower parts of the body, anæsthesis and paralysis of the lower extremities) imply no danger to life. Some relief at least can always be afforded by a proper attendance to the case. Lastly, the progress and the consequences of the affection are too little known to allow any estimate of its perilousness.

Balancing these considerations, we find the operation inadmissible, with the single exception adduced for swellings of the spleen in its normal place.

Dr. Piguacca, who has a splendid occasion to study the tumors of the milt, in the numerous cases occurring in the hospitals of Pavia, never saw (Ann. univ. Febr., Marzo, 1857,) any "manifest incommodation" from a wandering spleen. He considers the extirpation as an unwarranted procedure, as long as there are no considerable disturbances in the natural action of other organs; and this was never the case in the many patients that came under his observation.

Prof. Adelmann (Deutsche Klin., 17, 18, 1856,) has taken sides with Dr. Kuechler, defending the bold course adoped by the latter. Dr. Gurtt, (Deutsche Klin., 34, 1858,) and others, acknowledge the impregnable position of Simon, whose views appear scientifically correct.—*Schmidt's Jahrb.* 101, Feb. 1, 1859.—"*From the Cleveland Medical Gazette.*"

